# How Perceived Child-Friendly Communities Alleviate Adolescents’ Psychological Reactance

**DOI:** 10.3390/bs14100970

**Published:** 2024-10-19

**Authors:** Tiantian Liu, Shuge Xu, Lin Liu, Yue Chen, Wangwang Li

**Affiliations:** 1 School of Sociology, Central China Normal University, Wuhan 430079, China; eudora@mails.ccnu.edu.cn; 2 School of Government, Beijing Normal University, Beijing 100875, China; xushuge@mail.bnu.edu.cn (S.X.); 202221240008@mail.bnu.edu.cn (L.L.); yue.chen2@alumnimail.pepperdine.edu (Y.C.); 3 School of Sociology, Wuhan University, Wuhan 430072, China

**Keywords:** psychological reactance, child-friendly community, quality of life, propensity score matching

## Abstract

When children enter adolescence, their personality traits easily give rise to psychological reactance (PR). PR involves a desire for autonomy and independence, as well as an aversion to parental and social rules and restrictions. Factors that influence PR include physiological, familial, and social aspects. However, most studies on adolescent noncompliance have primarily focused on rebellious behavior. Little research has examined motivational state reactance, although its interaction with environmental perception can significantly affect adolescents’ lives. This study aimed to explore the relationship between the perceived friendliness of the community environment and cognitive processing at different quality of life (QoL) levels in relation to PR among adolescents. Propensity score matching was performed on a sample of 3546 adolescents, collected in Sanya, China. The results show that child-friendly communities (CFCs) had a significantly negative impact on adolescents’ PR levels. Additionally, QoL had a moderating effect, meaning that the higher the QoL, the stronger the role of a CFC in alleviating PR. In contrast to claims that PR is determined by temperament or character profiles, this study reveals the importance of environmental shaping through triadic reciprocal determinism and a focus on the key role of the community environment.

## 1. Introduction

Children tend to exhibit noncompliant and resistant behaviors as they enter adolescence. Children face many changes during adolescence, including gaining independence from their parents, discovering different facets of their identity, and dealing with hurdles in day-to-day life and at school [1]. Currently, academics generally use psychological reactance (PR) to explain adolescent noncompliance and resistance [2]. Notably, in contrast to rebellion, which is used to describe the resistant behavior of adolescents, an individual’s tendency toward reactance can be considered a personality trait [2]. Although for some time, reactance has been thought to be largely stable as a personality trait, recent research indicates that it can change through persistent intervention and stimulation [3]. It emerges gradually as the child enters adolescence, and consequently, its variability and sensitivity increase. This development can be partly attributed to important changes in the psychology of the child, which make individuals in this phase more susceptible to stimulation and influence from their families, schools, and community environments. Most studies on adolescent noncompliance have primarily focused on rebellious behavior, while relatively little research has examined motivational state reactance and its interaction with environmental perception.

Although most studies have examined reactance as an antecedent leading to adverse outcomes [4,5,6], from the perspective of personality and developmental psychology, they have ignored the antecedents of its formation and the influence of the environment. How do individuals structure their thinking about environmental issues, and how do these perceptions influence their personality traits? The theory of reciprocal determinism posits that environmental cognition ultimately influences individuals’ behavior by affecting their plans, expectations, self-regulation, and perceived behavioral control [7]. On the surface, noncompliance indicates conflict between teenagers and adults, but it is actually a reflection of the mismatch between teens’ environmental perceptions and their subjective needs [8]. In addition to family and school environments, child-friendly communities (CFCs) are an underinvestigated research topic in the field of adolescent development, and very little research has been conducted on the effects of a perceived CFC on adolescents’ reactance. Since the concept of “child-friendly cities” was first proposed by UNICEF and UN-Habitat in 1996, the construction of CFCs has increased around the world in response to the shrinking world of children [9]. The community is an important societal system in which youth can develop various connections. However, we know relatively little about how connections to the community promote the overall well-being of adolescents. As global urbanization continues to limit children’s free spaces and social opportunities, the establishment of CFCs has become crucial for nurturing adolescents’ positive development. Thus, we examined the motivational state of rebellion to understand how the community and environment shape PR.

Current research generally agrees that there is a significant relationship between PR and the subjective well-being of adolescents [10,11]. Quality of life (QoL) is a key indicator for assessing the well-being of adolescents, and it has attracted the attention of researchers from numerous fields of study in recent decades, especially those from the field of developmental psychology. Although the concept of health-related QoL has been frequently studied in adolescent psychiatry, the overall QoL of adolescents has not been associated with their development in general. QoL, as a subjective sense of well-being and a manifestation of living conditions, cannot be understood without the consideration of individual traits. It symbolizes a state of stable well-being and contentment for a period of time based on subjective experiences, reflecting individuals’ conscious perceptions and cognitive evaluations, while also influencing the way that they assess their environment [12]. Moreover, the COVID-19 pandemic has not been well investigated as a public health emergency that produced enormous challenges for people around the world and had significant consequences regarding the QoL of adolescents. Although the COVID-19 pandemic was officially declared over in every country in 2023, the question of whether its negative experiences (e.g., quarantine, class suspension, and isolation from society) imposed a burden on adolescents remains unanswered.

In summary, this study proposes the following two key questions: (1) Do perceived CFCs significantly alleviate adolescents’ PR? Generally, the measurement of CFCs can be divided into four dimensions, i.e., safety and protection (SP), educational diversification (ED), leisure and play (LP), and personal life (PL) [13]. Therefore, the first objective of this study was to explore the impact of the curtailment of perceived CFCs on adolescents’ PR. Specifically, the impacts of CFCs and their four dimensions (i.e., SP, ED, LP, and PL) on adolescents’ PR were examined. (2) Can QoL influence the relationship between perceived CFCs and adolescents’ PR? In particular, the second objective of this study was to examine the moderating role that QoL plays in the relationship between CFCs, including their four dimensions (i.e., SP, ED, LP, and PL), and adolescents’ PR.

### 1.1. Psychological Reactance in Puberty

The theory of PR was introduced in 1966 [14]. It holds that individuals perceive that they are free to perform various behaviors as they wish, and that, when this freedom is inhibited, they perceive it as aversive [14]. When their perceived freedom to act is threatened or constrained entirely, individuals are motivated to protect their perceived ability through cognitive or behavioral efforts, which may entail directly engaging in the prohibited behavior, refusing the threat, or applying the perceived ability in an alternative way [15]. Reactance refers to the motivational state that causes a personal response, and it consists of a combination of negative emotions and aversive cognitions [2]. It is a critical construct because it increases the possibility that individuals will become more engaged in the undesirable action in response to the forces attempting to reduce it.

Current theories of personality development generally suggest that adolescence is an important period for children to form and develop a sense of autonomy and self-identity; thus, during this period, adolescents are more likely to display a psychological state of reactance [2]. Specifically, children entering adolescence generally feel that their autonomy and freedom are threatened when they face a variety of coercive rules and restrictions that are beneficial for adapting to society and education [16,17]. By integrating PR theory and self-determination theory, Stijn Van Petegem et al. further concluded that adolescents’ psychological resistance manifests in the rejection of externally imposed rules, which is not only likely to incur emotional costs (internalizing problems) but also to cause undesirable behaviors (externalizing problems) [16]. Therefore, additional investigations of adolescents’ PR are necessary to reduce the accompanying internalizing and externalizing problems.

Currently, there is a large body of literature regarding the factors that cause adolescent PR. Previous studies have pointed out that negative peer relationships can foster adolescents’ psychological resistance, and that adolescents may mimic the reactance and disobedience of their peers in school, which may cause them to engage in negative behaviors and substance abuse, as well as to drop out of school [4,18,19]. Notably, recent research has considered PR a personality characteristic that may be shaped over time, gradually emerging and becoming particularly pronounced during adolescence [2]. This understanding highlights the adaptability of personality traits during adolescence. Jule Specht et al. synthesized various theories of personality development to point out that adolescents are more susceptible to strong changes caused by environmental factors than to those caused by adulthood [20]. Therefore, to reduce the PR of adolescents, it is crucial to create and maintain an environment that can have a positive impact on personality development. Adolescents are currently growing up under more supervision by adults, and they have fewer opportunities to explore their environment and learn how to solve life problems on their own [21]. In this environment, teenagers’ needs to develop autonomy and environmental competence are significantly suppressed, which leads to a higher possibility of state reactance and further overt resistance to parental and societal regulations [2,8]. Nevertheless, the environmental influences on adolescents’ PR have not been thoroughly investigated. Therefore, in the present study, we aimed to trace the motivational state of reactance to understand the “pre-cause (PR) of the precursor (rebellious behavior)” from a community–environmental perspective, which remains relatively underexplored in the research. We also sought to understand how QoL plays a role in determining the influences of the environment on PR in young adolescents.

### 1.2. The Relationship Between a Child-Friendly Community and Psychological Reactance

Scholars in the field of psychological cognition, as well as environmental psychology, particularly those who emphasize sociophysical environmental theories, have highlighted the potential interactive relationship between environmental perception and individual development, behavior, and well-being [7,22,23,24]. Given the rapid social changes and developments during the past 50 years or more, teenagers have experienced a gradual loss of behavioral freedom and an increase in adult supervision and control during childhood and early adolescence [21]. Increased parental control is specifically manifested as parents keeping their adolescents under a tighter rein because of concerns about traffic and stranger danger, which ultimately exacerbates the isolation of adolescents from community environments [21]. Meanwhile, by integrating PR theory and self-determination theory, Stijn Van Petegem et al. demonstrated that controlling parenting makes adolescents more likely to experience PR [16]. Therefore, increasing adolescents’ perception of the community environment is an important means of alleviating their PR.

In contrast to previous research that focused on the family, media, or other types of environments [25,26], the focus on CFCs emphasizes the children’s living environment and their perception of it. Specifically, CFCs are environments that improve the overall well-being of children, in which they not only have access to material necessities but can also have daily and spiritual life experiences; feel safe; learn and play; and feel that their voices are valued [9]. Studies have shown that subjective perceived environmental characteristics are closely related to actual psychological, behavioral, and health outcomes [27]. The measurement of community friendliness to youth can be divided into four sectors, i.e., SP, ED, LP, and PL [13]. To test the relationship between a conducive environment for adolescents and their level of PR, we hypothesize the following:

**H1.**  *When adolescents live in a community that is child-friendly, they demonstrate a lower level of PR.*

First, a safe and protective environment is crucial for adolescent development, offering opportunities for growth through experiences at home, at school, and in the neighborhood [21]. CFCs implement space-oriented policies that engage families, neighborhoods, social institutions, and the state in creating welcoming, accessible environments [21]. This approach balances protection with freedom, fostering adolescent exploration and self-efficacy. They promote independent mobility and transportation options, which not only enhance physical well-being but also improve the adolescents’ understanding of their local environment and community interactions [8]. In such settings, teenagers are granted appropriate levels of autonomy and responsibility. This freedom encourages independent thinking and the development of personal coping strategies while maintaining positive relationships with supportive adults. Consequently, adolescents in CFCs are likely to exhibit reduced PR.

**H1.1**  *As the level of SP in a CFC increases, the level of adolescents’ PR decreases.*

Second, in terms of education, the community can combine adolescents’ needs for growth and development. It can link adolescents to relevant expert resources and public welfare organizations according to their needs; offer them community education through community-themed lectures, knowledge classes, growth camps, and other activities; enrich their community entertainment; cultivate their learning abilities; and build a platform for their learning, communication, and all-around development [28]. Adolescents can engage in learning based on their interests and can achieve specific learning goals, which enables them to perceive freedom in learning. With increased perceived freedom, teenagers are likely to present lower PR because they perceive reduced control from adults.

**H1.2**  *As the ED in a CFC increases, the level of adolescents’ PR decreases.*

Third, providing adolescents with an open space for LP activities is a major function of CFCs [29]. This can also be referred to as the affordance and accessibility of a community [30]. Affordance involves the operational possibilities that may be perceived, discovered, changed, and employed by teenagers within their environment and neighborhoods. Accessibility is the possibility for adolescents to make use of these material resources. Adolescents whose communities include these settings find it easier to conduct loosely supervised, self-directed play activities with peers, friends, and even adults. With less parental and environmental control and regulation, teenagers can obtain higher perceived freedom and a lower degree of PR.

**H1.3**  *As the LP opportunities of a CFC increase, the level of adolescents’ PR decreases.*

Fourth, adolescents’ experiences of PL in their community are a direct criterion for gauging the level of the community’s child-friendliness. Many studies have shown that, when adolescents can stay away from violence and abuse, avoid contact with troubled people, and feel safe and protected in their neighborhoods, their subjective well-being tends to increase [10,13,30]. Hence, adolescents demonstrate a comparatively lower level of PR when their experiences with the community are relatively positive.

**H1.4**  *As the positive PL experience level of a CFC increases, adolescents’ level of PR decreases.*

### 1.3. The Moderating Effect of Quality of Life

Most investigations of adolescents’ QoL have focused on populations with specific health conditions and have measured their health-related QoL. However, QoL and health-related QoL are not interchangeable ideas, and the line differentiating them should not be blurred. QoL involves a comprehensive assessment of life functioning beyond health [31]. Hence, a consideration of QoL that is applicable to all adolescents is necessary. The measurement of general QoL has been formalized with the Personal Wellbeing Index, and it is based on a satisfaction scale. It refers to a state of well-being and contentment that remains relatively constant during a period of time [12]. The theoretical reason for this application can be explained by need theory. Need theory links living conditions to subjective well-being and suggests that people with higher-level living conditions are more likely to fulfill their basic needs, such as food, security, and health, which can lead to higher life satisfaction and happiness [32].

Indeed, QoL was found to be a crucial element affecting the psychological and emotional well-being of adolescents during the COVID-19 pandemic. Adolescents in families with a lower socioeconomic status and those who lived in limited spaces had a higher risk of suffering from psychological burdens and problems associated with mental well-being [1]. The impact of young people’s QoL on PR has been proven [33], but few studies have considered QoL as a moderator. PR reflects discontent with parental and social control [2]. When adolescents believe that their lives are satisfactory and happy, they recognize fewer threats to their perceived freedom and independence. Thus, we can consider that adolescents’ perceptions of QoL can influence their PR.

In addition to the influence of subjective assessments of well-being on adolescents’ personality traits, the individuals’ QoL shapes their perception of their surroundings, thus influencing their PR. Triadic reciprocal determinism theory, as proposed by Bandura in 1978 [34], suggests that people have distinct environmental responses based on their unique physiological traits and social attributes. Although the physical environment is consistent for all individuals, its perceived quality can vary greatly, depending on each person’s individual state. Environmental psychologists also contend that the way perception functions in a setting is a function of how people perceive their environment, but this perception, in turn, is dependent on subjectively defined information in this setting [24]. Therefore, interactions between adolescents with various levels of QoL and their perception of the environment may also differ. Although QoL has long existed as a condition and outcome variable, there have been attempts to use QoL as a moderating variable in the medical field [35]. Therefore, this study also regards QoL as a moderating variable between CFCs and PR. This approach attempts to supplement the research gap regarding the influence of adolescents’ QoL and their environment on PR after the outbreak of a global infectious disease. We propose the following hypothesis:

**H2.**  *The higher the QoL, the greater the impact of a perceived CFC on adolescents’ PR.*

We further subdivide Hypothesis 2 into four hypotheses.

Prior research has demonstrated the significance of assessing an individual’s well-being through the quality of their neighborhood [36]. For adolescents, feeling safe, secure, and protected are vital factors associated with their happiness and well-being [10]. Among the factors mentioned by adolescents as contributors to their sense of safety, two stand out as particularly critical, i.e., a secure place to live and the presence of responsible adults. Additionally, research by Kahneman [37] suggests that individuals’ current life state can influence their judgment of the environment. Happier individuals tend to hold more positively biased views of their surroundings and others, which facilitates their perception of a greater sense of security and belonging [38]. This underscores the intricate relationship between QoL and individuals’ perceptions of their surroundings. Therefore, we further hypothesize the following:

**H2.1**  *QoL enhances the influence of the level of SP in a CFC on the PR of adolescents.*

Adolescents highly value opportunities for community education. Engaging in discussions about their commonalities and differences is closely tied to their overall QoL [10]. Research also indicates that individuals who report higher life satisfaction tend to exhibit an increased curiosity and an interest in exploration [39]. This enables them to adapt more rapidly to the educational environment and to experience a greater sense of achievement. Additionally, living in a close-knit community and coexisting with neighbors from diverse backgrounds can offer adolescents a diverse, multidimensional, and multifaceted community education. The perception and choice of the educational environment play a crucial role in their social and cognitive development [40]. Therefore, we further hypothesize the following:

**H2.2**  *QoL enhances the influence of the level of ED provided by a CFC on the PR of adolescents.*

Moreover, a variety of games and entertainment options are important components of adolescents’ high QoL. Social games assist them in understanding the core principles of gaming and illustrate that specific norms underpin all social interactions. This contributes to the development of their awareness and the refinement of their social skills [29]. Simultaneously, playtime promotes positive emotional experiences and mitigates the prevalence of negative cognitive perceptions [41]. Interacting with peers also supports adolescents in achieving socialization and facilitates more effective integration into society. Therefore, we hypothesize the following:

**H2.3**  *QoL enhances the influence of LP opportunities in a CFC on the PR of adolescents.*

Finally, positive self-perception and experiences play an important role in the subjective well-being of adolescents [10,42]. Experiences of being treated well and valued by others, being safe from dangerous individuals, and feeling good about themselves are examples of things that teenagers say are related to their happiness. Given the strong correlation between positive personal experiences and subjective well-being, we propose the following hypothesis:

**H2.4**  *QoL enhances the influence of the level of positive PL experiences provided by a CFC on the PR of adolescents.*

## 2. Materials and Methods

### 2.1. The Data

This study was conducted in early 2023 in Sanya, Hainan Province, China, coinciding with the country’s official declaration of the end of COVID-19 prevention measures. As a multiethnic international tourist destination and China’s pioneer in establishing CFCs, Sanya offered an ideal setting to examine how such environments impact adolescent PR [43]. Our research team from Central China Normal University’s School of Sociology, in collaboration with the Sanya Education Bureau, conducted the study after obtaining approval from the university’s institutional review board.

We focused on students aged around 11 years, the average onset of puberty for Chinese teenagers [44]. We used the random sampling method. Specifically, three middle schools were randomly selected from each of the four administrative districts in Sanya. All seventh and eighth grade students in these schools responded to an online questionnaire, which mainly consists of two modules: basic information and a friendly community evaluation. The question types primarily include fill-in-the-blank and Likert-scale formats. We collected 3546 valid responses, achieving a 94.89% efficiency rate. The sample demographics were diverse, and 45.12% of participants were female, with an average age of 13.89 years. Public school students accounted for 78.00% of the participants. The quality of the schools attended varied, with 33.81% rated as good, 49.72% rated as average, and 16.47% rated as poor, based on Sanya Education Bureau assessments.

### 2.2. Measurements

#### 2.2.1. Psychological Reactance

Our study employed the Hong Psychological Reactance Scale (HPRS) to measure the dependent variable [45], PR. This 11-item scale, comprising four dimensions, uses a 5-point Likert-type format. Higher scores indicate greater reactance. The Chinese version, translated by Cao [46], demonstrated good reliability and validity, with a well-fitting model (χ^2^ (37) = 881.397, SRMR = 0.03, CFI = 0.96, TLI = 0.95, RMSEA = 0.08) and an overall Cronbach’s alpha of 0.926.

#### 2.2.2. Child-Friendly Community

For the independent variable, we utilized UNICEF’s Child Friendly Community Self-Assessment Tool for Adolescents [47,48], adapted for the Chinese context. Shen, Zhang, and Liu, along with the Chinese government, localized the international version and applied it in an assessment and survey of children’s rights in Changsha City, showing good reliability and validity in a Chinese sample [48]. This localized version includes 28 items across four dimensions, i.e., SP, ED, LP, and PL. For example, for SP, items include “I feel safe using buses” and “I know about the risks of using the Internet”. For ED, examples comprise “I get enough attention from my teachers” and “In my school, I have been taught about safe sex”. For LP, items include “In my community, I have places for play and sports” (detailed items can be found in the Supplementary Materials). Responses are recorded on a 5-point Likert scale ranging from strongly disagree (1) to strongly agree (5). We categorized participants into high- and low-level CFC groups based on the median score (low = 0 and high = 1). The model showed a good fit (χ^2^ (339) =4762.483, SRMR = 0.04, CFI = 0.91, TLI = 0.90, RMSEA = 0.06), with an overall Cronbach’s alpha of 0.946.

#### 2.2.3. Quality of Life

The moderating variable in this study was QoL. Subjective QoL was assessed using the Personal Wellbeing Index—School Children (PWI-SC) [49]. This seven-item scale measures subjective well-being across various life domains [13]. It has been proven to be effective in China [50]. The model fit was strong (χ^2^ (14) =267.335, SRMR = 0.02, CFI = 0.98, TLI = 0.97, RMSEA = 0.07), with a Cronbach’s alpha of 0.915.

#### 2.2.4. Covariates

We chose covariates for psychological reactance at three levels, i.e., individual factors, family factors, and school factors. Individual factors included age (measured in years as of 2023), gender (male or female), BMI (calculated as weight in kilograms divided by height in meters squared), ethnicity (whether the individual is an ethnic minority: 1 = Yes and 0 = No), sociability (measured using the Interpersonal Skills Scale [51] with a 5-point Likert scale, with higher scores indicating stronger social skills; Cronbach’s alpha = 0.879), and emotion regulation (assessed using the Emotion Regulation Questionnaire for Children and Adolescents [52,53], also using a 5-point Likert scale; Cronbach’s alpha = 0.881). Family factors included the presence of siblings (1 = Yes and 0 = No), home distance (ranging from 1 = Within Sanya city to 3 = Cities outside Hainan Province), and intimacy with parents (measured on a scale from 1 = Not close at all to 5 = Very close). School factors encompassed cadre rank (from 0 = No rank to 2 = School-level student cadre), school type (1 = Yes for public schools and 0 = No for private schools), and school quality (rated from 1 = Poor to 3 = Good).

### 2.3. Analytical Strategy

Causal relationships are central to social science research. The counterfactual model has enhanced the validity of causal inferences, with matching emerging as a key nonparametric method for creating counterfactual frameworks. This approach is particularly valuable when standard regression estimators may falter [54]. Propensity score matching, despite its limitations in addressing broader endogeneity, has gained prominence for estimating average treatment effects due to its reduced reliance on functional form assumptions [55].

Our study employed a two-step approach. First, we conducted a propensity score analysis to mitigate selection bias [56]. We designated individuals from low CFCs as the control group and those from high CFCs as the treatment group. Using the Stata 17.0 psmatch2 package, we estimated the average treatment effect on the treated (ATET). Our method involved 1:1 matching with replacement, a logit model estimation of propensity scores, and an application of the default caliper. The matching process incorporated all covariates, including the moderator, and it was restricted to the common support range. All variables were standardized. We assessed matching quality and performed a sensitivity analysis, following Rosenbaum’s framework, to evaluate the impact of potential confounders [57]. We also applied alternative matching techniques, including nearest-neighbor matching using the Mahalanobis distance and kernel matching for robustness.

In the second phase, we explored the potential moderating role of QoL through a multiple regression analysis. All variables were standardized, and the control variables remained consistent across the matching and regression procedures. This study performed heterogeneity tests to examine variations in causal and moderating effects across different subgroups.

## 3. Results

### 3.1. Descriptive Analysis Results

The descriptive analysis results of all variables are shown in Table 1. Overall, the mean value of PR was approximately 2.95 out of 5, the average QoL was approximately 4.27, the mean level of emotion regulation was approximately 3.3, and sociability was approximately 3.71, on average. The participants’ average level of intimacy with their parents was 4.2 out of 5. Ethnic minority participants comprised 38.64% of the sample, and 56.20% were locals. Nearly one-quarter of the sample held a student cadre position. A total of 78% attended public school.

This study also compared the differences between the high- and low-level CFCs. Before matching, communities with low levels of child-friendliness had a slightly higher average level of PR (3.05) than communities with high levels (2.85) of child-friendliness, whereas communities with high levels of child-friendliness had a higher average QoL (4.63) than communities with low levels of child-friendliness (3.88). Adolescents in high-level CFCs exhibited higher levels of emotion regulation, sociability, and intimacy with their parents. In these communities, a higher proportion of adolescents held student cadre positions (8.32% higher at the class level and 1.25% higher at the school level). In contrast, the low-level CFCs included 7.82% more local adolescents than the high-level CFCs and comprised 8.5% fewer adolescents who attended prestigious schools.

### 3.2. Multivariate Results

Regarding the multivariate results, we found that the normalized deviations for most variables in the matched groups were below 10%. T-tests generally failed to reject the null hypothesis of no systematic differences between the high and low CFCs. The matching process resulted in minimal sample loss, with fewer than 49 representative observations.

Table 2 presents the average treatment effects of CFCs and their subdimensions on adolescents’ PR. Our analysis revealed that high-level CFCs were associated with a 0.262 lower average level of PR than were low-level communities (*p* = 0.004, SD = 0.090). By examining the CFC subscales, we found that the PL, ED, and SP dimensions significantly reduced adolescents’ PR by 0.237, 0.203, and 0.170 units, respectively (*p* < 0.05). Contrary to our expectations, the LP dimension did not have a statistically significant effect on PR. These findings support H1, H1.1, H1.2, and H1.4, while H1.3 was not substantiated by our data.

To validate our findings, we conducted a sensitivity analysis and robustness checks on the matching results. The sensitivity analysis revealed no variables near the contour, suggesting the absence of unobservable effects on both the outcome and treatment variables. This outcome reinforced the significance of our results [57]. For robustness, we employed two additional matching methods (Table 3). The results of these alternative approaches largely maintained statistical significance, lending further credence to the reliability of our findings. These complementary analyses strengthen the overall validity of our study, demonstrating the consistency of our results across different methodological approaches.

### 3.3. Moderating Effects

Before conducting a multiple regression analysis, we addressed potential statistical issues. The Breusch–Pagan/Cook–Weisberg test for heteroskedasticity indicated the need for robust OLS estimations in all models, except for 2 and 6. Multicollinearity was assessed using variance inflation factors (VIFs), with mean VIF values between 1.23 and 1.33, well below concerning levels. The Shapiro–Wilk test revealed a non-normal distribution for some variables, prompting us to use the least squares method with adjusted standard errors.

**Figure 1:**
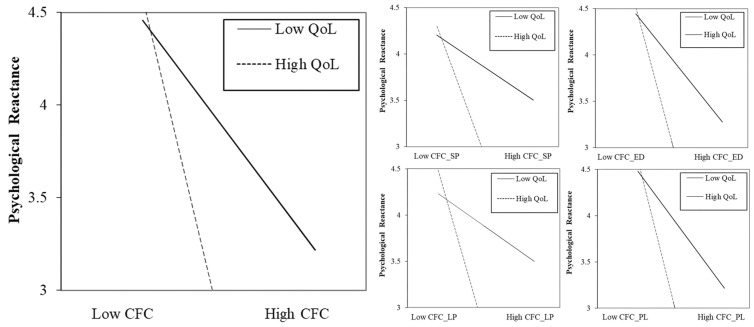
Moderating effects of QoL.

In most models in Table 4, the coefficients for the CFCs and their subscales are negative and significant, leading to the same conclusion as the one derived from the matching methods. To explore the moderating role of QoL on the relationship between CFCs and adolescents’ PR, we incorporated interaction terms into our regression models. Figure 1 and Table 4 present the comparative results of these analyses. Our findings revealed a significant moderating effect of QoL on the relationship between CFCs and PR (β = −0.043, *p* < 0.01). This effect was consistent across all four CFC subdimensions, supporting Hypothesis 2 and its sub-hypotheses (H2.1–H2.4). The strength of moderation varied among the CFC dimensions. QoL had a stronger enhancing effect on the inhibitory impact of CFCs focused on LP and PL (β = −0.048, *p* < 0.01). Conversely, the moderating effect was less pronounced in communities emphasizing SP (β = −0.035, *p* < 0.05).

In addition, some key patterns were revealed regarding the influence of the control variables on psychological reactance. Ethnicity showed a consistent positive effect, with ethnic minorities exhibiting higher reactance levels (e.g., 0.053, *p* < 0.01). Sociability strongly predicted higher reactance across all models (e.g., 0.275, *p* < 0.001), while better school quality, closer parent relationships, and stronger emotion regulation all significantly reduced reactance (e.g., school quality: −0.087, intimacy with parents: −0.086, and emotion regulation: −0.249; all *p* < 0.001).

### 3.4. Heterogeneity Tests

For heterogeneity tests, this study conducted subgroup analyses based on gender, region, ethnicity, and community type. We compared boys and girls, local and nonlocal residents, Han and ethnic minorities, and comprehensive and noncomprehensive community pilot areas.

**Figure 2:**
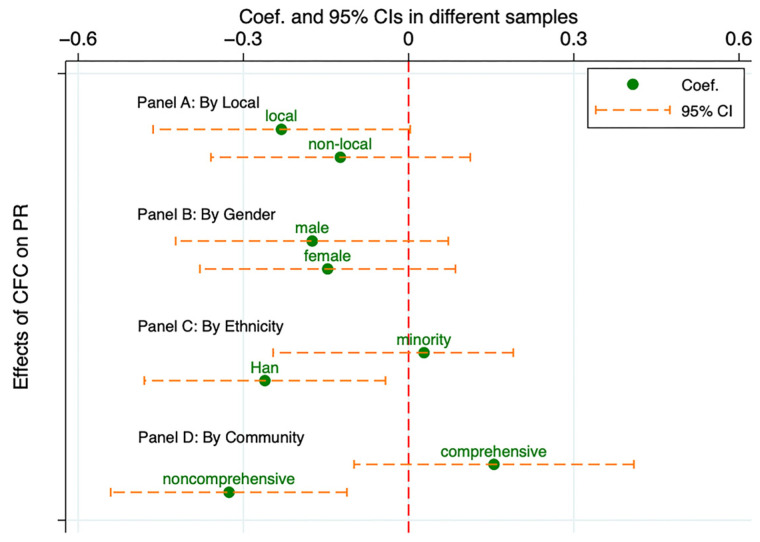
Heterogeneity test results for the estimation effect of CFCs on PR using propensity score matching.

**Figure 3:**
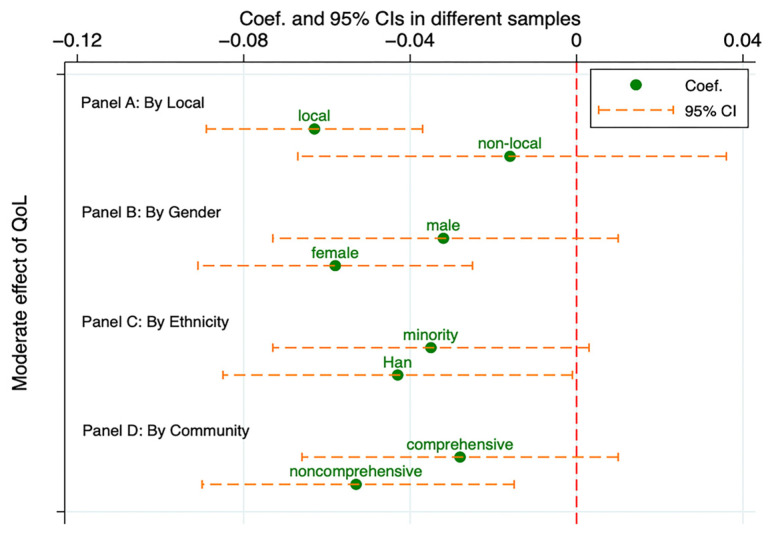
Heterogeneity test of the moderating effect of QoL on the effect of CFCs on PR.

Our findings revealed that CFCs had a more pronounced impact on Han ethnic children and those in noncomprehensive communities. In these subgroups, CFCs significantly mitigated PR. By examining the moderating effects across subgroups, we found notable differences only between local and nonlocal adolescents. For nonlocal youth, the relationship between CFCs and PR was not moderated by QoL. The enhancing effect of QoL was significant only among local adolescents. Other subgroup comparisons did not yield consistent or systematic differences. Figure 2 and Figure 3 provide a comprehensive overview of these analyses.

## 4. Discussion

In this study, we investigated the relationship between CFCs, PR, and QoL. Data were collected in Sanya in March 2023 (N = 3546). The findings of this study emphasize that friendly communities can mitigate overall adolescents’ reactance, especially across the dimensions of PL, ED, and SP. Although we hypothesized that LP would also mitigate PR, our data did not support this hypothesis. In short, H1, H1.1, H1.2, and H1.4 were supported, while H1.3 (LP) was not. Additionally, our findings demonstrate that QoL significantly moderated the relationship between the perception of a CFC and PR, validating H2, H2.1, H2.2, H2.3, and H2.4.

### 4.1. Theoretical Implications

Firstly, we found support for the hypothesis that CFCs can alleviate adolescents’ PR. Additionally, we verified the variability of adolescents’ PR in puberty as a personality trait from an environmental perspective, thereby advancing the study of adolescent resistance. Initially, this study demonstrates the dynamic variability of PR as a personality trait, expanding the scope of adolescent response research from a cognitive perspective. Previous studies have mostly considered that PR differs and stabilizes in adolescents with distinct temperament or character profiles [2]. We noticed subtle differences between PR and rebellious behavior, which is very different from adopting an attitude of systematic disobedience and rebellion. The distinction between PR and rebellious behavior can be analogized to the difference between cognitive or motivational factors and actual behavior. Therefore, we examined the motivational state of reactance to understand the “pre-cause (PR) of the precursor (rebellious behavior).” The findings clarify that the perceived environment could reduce adolescents’ PR, thus further demonstrating the variability of PR. Next, unlike previous discussions focused on the family and school climate [58], we underscore the role of the community environment in the formation of PR. This study reveals that CFCs can alleviate the formation of PR by teaching children relevant personal safety information, providing opportunities to learn on multiple levels, and offering children PL [59]. An earlier discussion pointed out that PR in adolescents mostly results from threats to autonomy and freedom due to coercive rules [16]. Therefore, this study demonstrates that CFCs can optimize children’s living space and improve their perception of the environment, thus reducing the negative psychological impact of controlling parenting. Finally, except for CFCs and their three dimensions of SP, ED, and PL, this study did not find evidence that the LP dimension reduces adolescents’ PR. Therefore, this study’s findings suggest that providing safe and protective environments, diverse educational opportunities, and positive personal life experiences is more important for adolescents’ mental health and well-being.

Secondly, we found support for the hypothesis that QoL can moderate the relationship between CFCs and PR. In this study, we took QoL as a moderator into theoretical consideration and applied triadic reciprocal determinism [34], thereby expanding the understanding of how the perceived environment shapes PR. Initially, considering adolescents’ post-pandemic well-being, we distinguished QoL from health-related quality and associated it with holistic development. Specifically, we define QoL as a conscious perceptual and cognitive evaluation of the community environment based on individual subjective experience, which can be used to measure adolescents’ well-being [12]. Next, our study demonstrates that QoL can play a moderating role, in addition to functioning as an outcome variable. Our findings demonstrate that QoL moderates the impact of CFCs and their four dimensions on adolescent PR. According to triadic reciprocal determinism, the perceived quality of the environment may be influenced by the state of the individual [24,34]. Thus, the moderating effect of QoL suggests that adolescents with higher QoL perceived greater environmental friendliness and a lower environmental threat, which enhanced their freedom and mitigated PR. This is especially important in the post-pandemic era, particularly for adolescents from families whose incomes and occupations were hard hit by the pandemic. To this end, the development of CFCs should take into account the existence of other factors, such as QoL. It may be necessary to implement projects aiming to improve adolescents’ QoL through institutions such as schools and communities to increase adolescents’ well-being and strengthen their positive environmental perceptions.

Finally, we considered some factors that may influence the effect of CFCs on adolescents’ PR from individual and environmental perspectives. Initially, our findings suggest that Han Chinese are more likely than ethnic minorities to be influenced by their community in terms of their PR. A possible explanation for this is that ethnicity, as an individual’s collective identity, has a lasting and subtle influence on individual psychology that is difficult to change through environmental shaping. Therefore, the marginal status of ethnic minorities in the shaping of CFCs makes their access to targeted policy practices less likely, which leads to their PR and causes them to rely on family education methods. Next, in the comparison between locals and nonlocals, we found that there may be community segregation or integration issues that limit the community’s effect on improving nonlocal adolescents’ PR. This point can perhaps be explained by the ingroup–outgroup effect noted in social psychology. Local adolescents’ stronger sense of belonging to the community makes it easier for them to seek help from the community, in addition to family and relatives. Additionally, due to the impact of COVID-19 and its isolation policies, long-term stable community relationships led to differences in individual QoL and community environment perceptions for local adolescents.

In a context in which global urbanization reduces adolescents’ activity spaces, reduced opportunities to interact with peers lead to a greater risk of heightened PR in adolescents [4]. Furthermore, the prolonged three-year pandemic indelibly impacted people’s lives and environments, and the class suspension and isolation from society that resulted from the pandemic significantly affected adolescents’ QoL. In this context, it is necessary to comprehensively explore how supportive communities shape children’s psychological development. Therefore, although our previous research demonstrated the positive psychological effects of CFCs in educationally disadvantaged youth [13], we expanded our study to general adolescents in order to achieve a more comprehensive understanding of the relationship between CFCs and PR. In summary, we gained new theoretical insights indicating that CFCs can directly alleviate adolescents’ PR, and that QoL can effectively moderate this relationship.

### 4.2. Practical Implications

A CFC is a community planning approach that supports children’s rights and welfare in everyday and emergency situations. CFCs can promote the growth of children and adolescents in several ways. First, the design of the physical environment and operations should prioritize safety factors. Community service workers can contribute to safety efforts by sharing knowledge with parents and children and encouraging active participation in creating a secure environment. Second, community-based nonformal education can offer organized learning opportunities, particularly for children and youth with special needs, or provide additional support for school-going children. We advise the adoption of child-centered learning methods, as well as allowing children to connect and interact socially as much as possible. Third, the acquisition of health and life skills is an essential aspect of a CFC, and include activities such as promoting immunization; offering health screenings; providing diet guidance and exercise instructions; and delivering guidance on sexual health, drug, and alcohol impacts and HIV knowledge in culturally acceptable contexts. Finally, all services should be based on inclusive and nondiscriminatory approaches to ensure equal access for children and youth from diverse backgrounds.

Furthermore, greater efforts should focus on elevating youths’ QoL. A holistic, youth-oriented pandemic recovery plan should focus on reinforcing mental health, restoring social bonds, redressing educational setbacks, encouraging healthy behaviors, creating job and training opportunities, and providing families with economic and emotional aid. This multidimensional approach seeks to build resilience and improve well-being in the youth population.

### 4.3. Limitations and Further Research

There are still many limitations to be overcome, thus providing guidance for future research. First, due to the COVID-19 pandemic, the data in this study could only be collected at its end and were not observed over a long period of time. A longitudinal research perspective is therefore lacking. Thus, future research may continue the observation in order to further validate the results of this study. Second, the reliability and validity of some of the measurement scales have not previously been examined with Chinese samples, which may be a limitation of the methodology. Although numerous factors are related to adolescents’ PR, because our research topic was the influence of CFCs on adolescents’ PR, we focused on the influence of community and environmental factors on adolescents’ PR and did not pay attention to other factors, which could be included in similar studies in the future. Third, the concept of CFCs was introduced into China in 2019 and has been in development for only four years thus far; thus, its construction in Chinese cities is lagging. Notably, our data were drawn from a sample in Sanya, China, which may pose limitations regarding the sample’s generalizability. Therefore, future research may consider other regions or countries with different levels of development of CFCs, which would verify whether there is any particularity in the results of this study.

## 5. Conclusions

As a common motivational personality trait in adolescents during puberty, PR can provide clues to their motivations and behaviors. Our research addresses the limitations of traditional regression analyses and confirms the protective role of community environments in shaping PR. As global urbanization reduces children’s activity spaces and restricts prosocial behaviors, the need to establish CFCs in order to mitigate adolescents’ disobedience has become increasingly apparent. Our research focused on adolescents’ QoL in the post-pandemic era to examine how well-being as a holistic state yields differences in perceptions of the changing environment and how individual states interact with supportive community environments to impact psychological growth. Continuously monitoring and effectively improving adolescents’ QoL should be a shared responsibility of families and society. This research provides a new perspective for studies on adolescents’ psychological development and CFC building.

## Figures and Tables

**Table 1:** Descriptive characteristics of the participants according to the CFC before and after propensity score matching.

Variables	Total		Unmatched				Matched			
			High-Level CFCs		Low-Level CFCs		High-Level CFCs		Low-Level CFCs	
	Mean (%)	SD	Mean (%)	SD	Mean (%)	SD	Mean (%)	SD	Mean (%)	SD
PR (1–5)	2.95	0.89	2.85	1.05	3.05	0.66	2.91	0.83	2.98	0.71
Age	13.89	0.75	13.87	0.74	13.91	0.77	13.90	0.76	13.90	0.83
Gender										
Female	45.12		44.19		46.12		45.21		44.96	
Male	54.88		55.81		53.88		54.79		55.04	
BMI	18.31	3.98	18.33	4.10	18.28	3.86	18.31	4.06	18.37	3.87
Ethnicity										
Han	61.36		67.43		54.87		61.88		57.88	
Minority	38.64		32.57		45.13		38.12		42.12	
Siblings	0.85	0.36	0.84	0.37	0.87	0.34	0.87	0.34	0.85	0.36
QoL (1–5)	4.27	0.67	4.63	0.48	3.88	0.62	4.19	0.53	4.21	0.55
Emotion regulation (1–5)	3.30	0.54	3.45	0.55	3.15	0.47	3.28	0.53	3.25	0.46
Sociability (1–5)	3.71	0.73	4.00	0.69	3.39	0.62	3.59	0.64	3.61	0.55
Intimacy with parents (1–5)	4.20	0.86	4.34	0.82	4.04	0.88	4.16	0.90	4.22	0.85
Cadre rank										
No rank	74.68		70.05		79.63		75.35		74.87	
Class-level student cadre	23.86		27.88		19.56		23.23		23.36	
School-level student cadre	1.47		2.07		0.82		1.42		1.77	
Home distance										
Within Sanya (local)	56.20		52.43		60.25		55.32		60.35	
Other cities within Hainan	22.65		25.86		19.21		23.58		19.47	
Cities outside Hainan	21.15		21.71		20.55		21.10		20.18	
School type										
Private school	22.00		23.24		20.67		22.70		20.71	
Public school	78.00		76.76		79.33		77.30		79.29	
School quality										
Poor	16.47		14.40		18.68		16.49		18.41	
Average	49.72		47.68		51.90		50.53		49.20	
Good	33.81		37.92		29.42		32.98		32.39	
Total	3546		1833		1713		564		565	

**Table 2:** Average treatment effects of CFC and its subdimensions on adolescents’ PR.

Treatment Dimensions	Estimation	Robust Std. Error	*z*	*p* > *z*	[95% Confidence Interval]	
CFC	−0.262	0.090	−2.92	0.004	−0.437	−0.086
SP	−0.170	0.071	−2.40	0.016	−0.309	−0.031
ED	−0.203	0.079	−2.58	0.010	−0.358	−0.049
LP	0.051	0.082	0.620	0.534	−0.109	0.211
PL	−0.237	0.110	−2.15	0.032	−0.453	−0.021

**Table 3:** Robustness checks.

Treatment Dimension	Propensity Score Matching	Nearest-Neighbor Matching	Kernel Matching
CFC	−0.262 **	−0.164 ***	−0.233 **
	(0.090)	(0.043)	(0.088)
SP	−0.170 *	−0.129 **	−0.132 *
	(0.071)	(0.047)	(0.065)
ED	−0.203 *	−0.199 ***	−0.177 *
	(0.079)	(0.041)	(0.083)
LP	0.051	−0.038	0.002
	(0.082)	(0.047)	(0.078)
PL	−0.237 *	−0.173 ***	−0.211 **
	(0.110)	(0.050)	(0.078)

Note: robust std. err. in parentheses. *** *p* < 0.001; ** *p* < 0.01; * *p* < 0.05.

**Table 4:** Moderating effects of QoL.

Variable	DV: PR									
	CFC		CFC_SP		CFC_ED		CFC_LP		CFC_PL	
	Model 1	Model 2	Model 3	Model 4	Model 5	Model 6	Model 7	Model 8	Model 9	Model 10
IVs:	−0.045	−0.057 *	−0.017	−0.018	−0.052 *	−0.063 **	0.008	−0.002	−0.059 **	−0.076 ***
	(0.024)	(0.025)	(0.019)	(0.019)	(0.022)	(0.023)	(0.021)	(0.021)	(0.021)	(0.022)
Moderator: QoL	−0.079 **	−0.109 ***	−0.099 ***	−0.119 ***	−0.078 ***	−0.104 ***	−0.110 ***	−0.139 ***	−0.070 **	−0.101 ***
	(0.024)	(0.025)	(0.022)	(0.023)	(0.023)	(0.024)	(0.023)	(0.023)	(0.024)	(0.025)
Interaction: IV × moderator		−0.043 **		−0.035 *		−0.041 **		−0.048 **		−0.048 **
		(0.015)		(0.018)		(0.015)		(0.015)		(0.014)
Age	0.025	0.027	0.025	0.026	0.025	0.026	0.025	0.026	0.026	0.028
	(0.016)	(0.016)	(0.016)	(0.016)	(0.016)	(0.016)	(0.016)	(0.016)	(0.016)	(0.016)
Gender	0.022	0.024	0.022	0.024	0.021	0.023	0.023	0.026	0.021	0.023
	(0.032)	(0.031)	(0.032)	(0.031)	(0.032)	(0.031)	(0.032)	(0.031)	(0.032)	(0.031)
BMI	−0.014	−0.015	−0.014	−0.015	−0.014	−0.015	−0.015	−0.015	−0.013	−0.013
	(0.017)	(0.017)	(0.017)	(0.017)	(0.017)	(0.017)	(0.017)	(0.017)	(0.017)	(0.017)
Ethnicity	0.053 **	0.050 **	0.054 **	0.053 **	0.053 **	0.051 **	0.055 **	0.052 **	0.051 **	0.048 **
	(0.037)	(0.037)	(0.037)	(0.037)	(0.037)	(0.036)	(0.037)	(0.036)	(0.037)	(0.037)
Siblings	0.024	0.023	0.024	0.025	0.024	0.023	0.025	0.023	0.024	0.022
	(0.044)	(0.044)	(0.044)	(0.044)	(0.044)	(0.044)	(0.044)	(0.044)	(0.044)	(0.044)
Cadre rank	−0.058 ***	−0.059 ***	−0.059 ***	−0.061 ***	−0.058 ***	−0.059 ***	−0.060 ***	−0.061 ***	−0.059 ***	−0.060 ***
	(0.016)	(0.016)	(0.016)	(0.016)	(0.016)	(0.016)	(0.016)	(0.016)	(0.016)	(0.016)
Home distance	−0.016	−0.016	−0.017	−0.016	−0.015	−0.016	−0.017	−0.017	−0.015	−0.015
	(0.017)	(0.017)	(0.017)	(0.017)	(0.017)	(0.017)	(0.017)	(0.017)	(0.017)	(0.017)
Sociability	0.268 ***	0.276 ***	0.262 ***	0.266 ***	0.268 ***	0.274 ***	0.257 ***	0.264 ***	0.268 ***	0.275 ***
	(0.023)	(0.024)	(0.023)	(0.023)	(0.023)	(0.023)	(0.023)	(0.023)	(0.023)	(0.023)
School type	−0.032 *	−0.03	−0.032 *	−0.031	−0.032 *	−0.031	−0.033 *	−0.031	−0.034 *	−0.032 *
	(0.039)	(0.039)	(0.039)	(0.039)	(0.039)	(0.039)	(0.039)	(0.039)	(0.039)	(0.039)
School quality	−0.086 ***	−0.087 ***	−0.087 ***	−0.087 ***	−0.087 ***	−0.086 ***	−0.087 ***	−0.089 ***	−0.086 ***	−0.087 ***
	(0.018)	(0.018)	(0.018)	(0.018)	(0.018)	(0.018)	(0.018)	(0.018)	(0.018)	(0.018)
Intimacy with parents	−0.090 ***	−0.086 ***	−0.090 ***	−0.086 ***	−0.090 ***	−0.086 ***	−0.088 ***	−0.084 ***	−0.090 ***	−0.086 ***
	(0.018)	(0.017)	(0.018)	(0.018)	(0.018)	(0.017)	(0.018)	(0.018)	(0.018)	(0.017)
Emotion regulation	−0.252 ***	−0.247 ***	−0.253 ***	−0.249 ***	−0.250 ***	−0.247 ***	−0.253 ***	−0.248 ***	−0.253 ***	−0.249 ***
	(0.018)	(0.018)	(0.018)	(0.018)	(0.018)	(0.018)	(0.018)	(0.018)	(0.018)	(0.018)
Comprehensive community	0.018	0.018	0.018	0.018	0.018	0.018	0.017	0.017	0.018	0.018
	(0.032)	(0.032)	(0.032)	(0.032)	(0.032)	(0.032)	(0.032)	(0.032)	(0.032)	(0.032)
Observations	3546	3546	3546	3546	3546	3546	3546	3546	3546	3546
VIF	1.33	1.33	1.23	1.23	1.29	1.3	1.26	1.27	1.31	1.32
B-P/C-W Test *p*	0.000	0.164	0.000	0.006	0.000	0.112	0.000	0.032	0.000	0.064
*R* ^2^	0.132	0.137	0.132	0.134	0.133	0.137	0.131	0.137	0.133	0.139
Adjusted *R*^2^	0.129	0.133	0.128	0.13	0.129	0.133	0.128	0.133	0.129	0.135

Note: Standard errors in parentheses. *** *p* < 0.001; ** *p* < 0.01; * *p* < 0.05.

## Data Availability

The data presented in this study are available on request from the corresponding author. The data are not publicly available due to privacy and ethical restrictions.

## Supplementary Materials

The following supporting information can be downloaded at: https://www.mdpi.com/article/10.3390/bs14100970/s1, Table S1: Child Friendly Community Scale (Chinese Version); Table S2: Cronbach’s alpha results of Child Friendly Community Scale; Table S3: Cronbach’s alpha results of Hong Psychological Reactance Scale; Table S4: Cronbach’s alpha results of Personal Wellbeing Index—School Children; Table S5: Cronbach’s alpha results of Emotion Regulation Questionnaire for Children and Adolescents Scale; Table S6: Cronbach’s alpha results of Interpersonal Skills Scale; Table S7: Balancing hypothesis test showing the variables’ characteristics before and after matching (CFC); Table S8: Balancing hypothesis test showing the variables’ characteristics before and after matching (CFCSP); Table S9: Balancing hypothesis test showing the variables’ characteristics before and after matching (CFCED); Table S10: Balancing hypothesis test showing the variables’ characteristics before and after matching (CFCLP); Table S11: Balancing hypothesis test showing the variables’ characteristics before and after matching (CFCPL).
