# Supplementary material for: How Perceived Child-Friendly Communities Alleviate Adolescents’ Psychological Reactance
Source: Behav Sci (Basel). 2024 Oct 19;14(10):970. doi: 10.3390/bs14100970 (PMC11504971; doi:10.3390/bs14100970)
Supplement: Supplementary file 1 [file behavsci-14-00970-s001.zip › behavsci-3188188-supplementary.pdf]

Table S1 Child Friendly Community Scale (Chinese Version)

Dimension	No.	Questions
**Security Protection**	1	I feel safe using buses or other public vehicles
	2	It is safe for me to walk and cycle in my community
	3	If I feel in danger, I know where to report it and get help
	4	If there is a hazard in my community, I know what to do
	5	I know about the risks of using the Internet
**Education**	1	Boys and girls are treated the same way in school
	2	I get enough attention from my teachers when I need it
	3	In my school I learn about being healthy
	4	In my school I learn how to protect the environment
	5	In my school I have been taught what my rights and the Convention on the Rights of the Child
	6	In school have been taught about safe sex
	7	My ideas are listened to by teachers in school
	8	There is free time in my school to play games and sports, rest and spend time with friends
	9	In my school there are adults who I feel safe talking to about my problems and feelings
	10	At my school, I am disciplined without being physically hurt
	11	I have opportunities to give my opinion about school decisions
**Leisure & Play**	1	In my community I have places for play, games and sports
	2	I have enough time to play, rest and enjoy myself
	3	The places for play in my community can also be used by children with physical disabilities
	4	There are places in my community where I can be in contact with nature
	5	I participate in or observe activities of cultures different from my own
	6	I participate in projects, groups or programs with other children or youth outside of school

**Personal Life**	1	I feel safe at home
	2	I feel safe from being bullied by other children
	3	I am free from drugs in the community
	4	I feel protected from being taken away by a stranger
	5	I feel safe from violence and abuse (Abuse could be verbal, physical or sexual)
	6	Children respect each other at my school without risk of being hassled or bullied

Note. The scale is developed by United Nations International Children's Emergency Fund with the Chinese government.

Table S2 Cronbach's alpha results of Child Friendly Community Scale.

Item	Observation	Sign	Item-test correlation	Item-rest correlation	Interitem covariance	alpha
1	3546	+	0.535	0.491	0.333	0.946
2	3546	+	0.508	0.459	0.333	0.946
3	3546	+	0.589	0.555	0.334	0.945
4	3546	+	0.576	0.538	0.333	0.945
5	3546	+	0.525	0.478	0.333	0.946
6	3546	+	0.612	0.576	0.331	0.945
7	3546	+	0.683	0.654	0.329	0.944
8	3546	+	0.712	0.689	0.332	0.944
9	3546	+	0.720	0.698	0.332	0.944
10	3546	+	0.643	0.607	0.328	0.944
11	3546	+	0.557	0.511	0.330	0.946
12	3546	+	0.700	0.669	0.326	0.944
13	3546	+	0.618	0.576	0.327	0.945
14	3546	+	0.706	0.675	0.326	0.944
15	3546	+	0.635	0.594	0.327	0.945
16	3546	+	0.706	0.677	0.327	0.944
17	3546	+	0.668	0.631	0.326	0.944
18	3546	+	0.691	0.661	0.328	0.944
19	3546	+	0.632	0.597	0.330	0.944
20	3546	+	0.656	0.630	0.334	0.944
21	3546	+	0.650	0.615	0.329	0.944
22	3546	+	0.617	0.578	0.329	0.945
23	3546	+	0.640	0.610	0.333	0.944
24	3546	+	0.713	0.685	0.327	0.944
25	3546	+	0.703	0.675	0.329	0.944
26	3546	+	0.699	0.670	0.328	0.944
27	3546	+	0.671	0.636	0.326	0.944
28	3546	+	0.696	0.666	0.327	0.944
Test scale					0.330	0.946

Table S3: Cronbach's alpha results of Hong Psychological Reactance Scale.

Item	Observation	Sign	Item-test correlation	Item-rest correlation	Interitem covariance	alpha
1	3546	+	0.782	0.730	0.734	0.918
2	3546	+	0.616	0.543	0.784	0.926
3	3546	+	0.790	0.735	0.721	0.918
4	3546	+	0.806	0.758	0.725	0.917
5	3546	+	0.755	0.696	0.739	0.920
6	3546	+	0.785	0.731	0.728	0.918

7	3546	+	0.756	0.696	0.738	0.920
8	3546	+	0.816	0.772	0.728	0.917
9	3546	+	0.709	0.644	0.753	0.922
10	3546	+	0.747	0.682	0.731	0.921
11	3546	+	0.779	0.722	0.726	0.919
Test scale					0.737	0.926

Table S4: Cronbach's alpha results of Personal Wellbeing Index—School Children.

Item	Observation	Sign	Item-test correlation	Item-rest correlation	Interitem covariance	alpha
1	3546	+	0.807	0.729	0.409	0.903
2	3546	+	0.801	0.723	0.414	0.904
3	3546	+	0.787	0.698	0.411	0.906
4	3546	+	0.801	0.729	0.420	0.903
5	3546	+	0.824	0.757	0.413	0.900
6	3546	+	0.854	0.793	0.398	0.896
7	3546	+	0.826	0.750	0.399	0.901
Test scale					0.409	0.915

Table S5: Cronbach's alpha results of Emotion Regulation Questionnaire for Children and Adolescents Scale.

Item	Observation	Sign	Item-test correlation	Item-rest correlation	Interitem covariance	alpha
1	3546	+	0.664	0.574	0.618	0.871
2	3546	+	0.705	0.618	0.600	0.868
3	3546	+	0.738	0.665	0.601	0.865
4	3546	+	0.737	0.666	0.604	0.865
5	3546	+	0.761	0.696	0.599	0.863
6	3546	+	0.772	0.708	0.593	0.862
7	3546	+	0.599	0.490	0.630	0.878
8	3546	+	0.656	0.551	0.608	0.874
9	3546	+	0.675	0.579	0.606	0.871
10	3546	+	0.670	0.571	0.607	0.872
Test scale					0.607	0.881

Table S6: Cronbach's alpha results results of Interpersonal Skills Scale.

Item	Observation	Sign	Item-test correlation	Item-rest correlation	Interitem covariance	alpha
1	3546	+	0.759	0.683	0.457	0.861
2	3546	+	0.747	0.674	0.467	0.862
3	3546	+	0.731	0.658	0.475	0.864
4	3546	+	0.656	0.547	0.475	0.873
5	3546	+	0.697	0.602	0.468	0.868

6	3546	+	0.563	0.422	0.493	0.886
7	3546	+	0.746	0.657	0.451	0.863
8	3546	+	0.788	0.717	0.447	0.858
9	3546	+	0.782	0.710	0.450	0.859
Test scale					0.465	0.879

Table S7: Balancing hypothesis test showing the variables' characteristics before and after matching (CFC).

Variables	Umatched / Matched	Mean Treated Group	Mean Control Group	Bias (%)	t-Value	p-Value
Age	U	-0.021	0.022	-4.3	-1.29	0.197
	M	-0.021	0.120	-14.2	-4.03	0.000
Gender	U	0.558	0.539	3.9	1.15	0.249
	M	0.560	0.550	2.1	0.63	0.527
BMI	U	0.007	-0.007	1.4	0.41	0.683
	M	0.004	-0.042	4.6	1.42	0.156
Ethnicity	U	0.326	0.451	-26.0	-7.74	0.000
	M	0.327	0.334	-1.2	-0.39	0.699
Siblings	U	0.836	0.867	-8.8	-2.60	0.009
	M	0.837	0.858	-6.0	-1.80	0.073
Cadre rank	U	0.110	-0.118	23.0	6.83	0.000
	M	0.095	0.187	-9.2	-2.50	0.013
Home distance	U	0.054	-0.058	11.1	3.32	0.001
	M	0.052	0.037	1.5	0.44	0.661
Sociability	U	0.405	-0.433	92.4	27.43	0.000
	M	0.398	0.412	-1.5	-0.43	0.668
School type	U	0.768	0.793	-6.2	-1.85	0.064
	M	0.769	0.819	-12.2	-3.77	0.000
School quality	U	0.090	-0.096	18.7	5.55	0.000
	M	0.084	0.032	5.3	1.54	0.123
Intimacy with parents	U	0.169	-0.180	35.4	10.54	0.000
	M	0.166	0.040	12.8	3.79	0.000
Emotion regulation	U	0.267	-0.286	57.7	17.12	0.000
	M	0.258	0.128	13.5	4.11	0.000
Quality of life	U	0.542	-0.580	135.1	40.37	0.000
	M	0.539	0.529	1.3	0.44	0.661

Note. U=Unmatched, M=Matched

Table S8: Balancing hypothesis test showing the variables' characteristics before and after matching (CFCSP).

Variables	Umatched / Matched	Mean Treated Group	Mean Control Group	Bias (%)	t-Value	p-Value
Age	U	0.010	-0.015	2.5	0.72	0.470
	M	0.009	0.066	-5.7	-1.88	0.061
Gender	U	0.563	0.527	7.4	2.15	0.032
	M	0.564	0.541	4.4	1.45	0.147
BMI	U	0.032	-0.048	8.0	2.32	0.021
	M	0.029	0.049	-2.0	-0.64	0.523
Ethnicity	U	0.326	0.477	-31.1	-9.13	0.000
	M	0.326	0.284	8.7	3.00	0.003
Siblings	U	0.838	0.870	-9.2	-2.65	0.008
	M	0.838	0.828	2.8	0.86	0.388
Cadre rank	U	0.084	-0.126	21.4	6.16	0.000
	M	0.081	0.074	0.7	0.21	0.835
Home distance	U	0.063	-0.095	16.0	4.64	0.000
	M	0.064	0.046	1.8	0.58	0.562
Sociability	U	0.269	-0.403	71.7	20.76	0.000
	M	0.268	0.240	3.0	0.96	0.338
School type	U	0.779	0.781	-0.5	-0.16	0.874
	M	0.779	0.759	4.8	1.53	0.126
School quality	U	0.092	-0.138	23.1	6.77	0.000
	M	0.092	0.207	-11.5	-3.87	0.000
Intimacy with parents	U	0.061	-0.091	15.2	4.44	0.000
	M	0.061	-0.091	15.2	4.80	0.000
Emotion regulation	U	0.141	-0.211	36.2	10.42	0.000
	M	0.139	0.000	14.3	4.51	0.000
Quality of life	U	0.281	-0.422	73.6	21.87	0.000
	M	0.281	0.232	5.1	1.88	0.061

Note. U=Unmatched, M=Matched

Table S9: Balancing hypothesis test showing the variables' characteristics before and after matching (CFCED).

Variables	Umatched / Matched	Mean: Treated Group	Mean: Control Group	Bias (%)	t-Value	p-Value
Age	U	-0.030	0.029	-5.9	-1.76	0.079
	M	-0.028	-0.029	0.2	0.05	0.964
Gender	U	0.561	0.537	4.9	1.47	0.143
	M	0.562	0.532	6.1	1.80	0.072
BMI	U	-0.003	0.003	-0.7	-0.20	0.844
	M	-0.003	0.063	-6.5	-1.84	0.066
Ethnicity	U	0.335	0.436	-20.8	-6.19	0.000
	M	0.335	0.335	0.1	0.04	0.971
Siblings	U	0.835	0.867	-8.9	-2.66	0.008
	M	0.835	0.785	14.0	3.76	0.000
Cadre rank	U	0.106	-0.103	20.9	6.24	0.000
	M	0.102	0.241	-13.9	-3.70	0.000
Home distance	U	0.055	-0.054	10.9	3.24	0.001
	M	0.054	0.181	-12.7	-3.57	0.000
Sociability	U	0.407	-0.395	87.5	26.06	0.000
	M	0.405	0.412	-0.8	-0.23	0.817
School type	U	0.767	0.793	-6.1	-1.82	0.068
	M	0.768	0.755	3.2	0.91	0.361
School quality	U	0.091	-0.089	18.1	5.39	0.000
	M	0.089	0.032	5.8	1.65	0.099
Intimacy with parents	U	0.169	-0.164	33.7	10.04	0.000
	M	0.167	0.163	0.5	0.15	0.884
Emotion regulation	U	0.289	-0.281	59.5	17.72	0.000
	M	0.284	0.252	3.4	1.02	0.310
Quality of life	U	0.531	-0.517	123.3	36.64	0.000
	M	0.531	0.511	2.3	0.76	0.445

Note. U=Unmatched, M=Matched

Table S10: Balancing hypothesis test showing the variables' characteristics before and after matching (CFCLP).

Variables	Umatched / Matched	Mean Treated Group	Mean Control Group	Bias (%)	t-Value	p-Value
Age	U	-0.033	0.025	-5.8	-1.72	0.086
	M	-0.031	0.052	-8.3	-2.29	0.022
Gender	U	0.567	0.535	6.4	1.89	0.059
	M	0.567	0.555	2.4	0.65	0.513
BMI	U	0.002	-0.001	0.3	0.08	0.936
	M	0.002	-0.008	1.0	0.29	0.773
Ethnicity	U	0.337	0.425	-18.2	-5.36	0.000
	M	0.337	0.324	2.7	0.77	0.444
Siblings	U	0.833	0.865	-9.0	-2.66	0.008
	M	0.833	0.821	3.3	0.86	0.391
Cadre rank	U	0.102	-0.079	18.0	5.36	0.000
	M	0.101	0.235	-13.3	-3.26	0.001
Home distance	U	0.078	-0.060	13.7	4.06	0.000
	M	0.077	0.098	-2.1	-0.58	0.565
Sociability	U	0.453	-0.349	86.6	25.78	0.000
	M	0.451	0.434	1.8	0.50	0.620
School type	U	0.780	0.780	-0.2	-0.07	0.947
	M	0.780	0.782	-0.5	-0.13	0.896
School quality	U	0.079	-0.061	14.0	4.13	0.000
	M	0.079	0.063	1.5	0.41	0.680
Intimacy with parents	U	0.127	-0.098	22.7	6.69	0.000
	M	0.134	0.090	4.4	1.23	0.218
Emotion regulation	U	0.260	-0.200	46.9	13.97	0.000
	M	0.260	0.165	9.7	2.64	0.008
Quality of life	U	0.571	-0.440	118.6	34.49	0.000
	M	0.570	0.557	1.5	0.49	0.626

Note. U=Unmatched, M=Matched

Table S11: Balancing hypothesis test showing the variables' characteristics before and after matching (CFCPL).

Variables	Umatched / Matched	Mean: Treated Group	Mean: Control Group	Bias (%)	t-Value	p-Value
Age	U	-0.014	0.012	-2.6	-0.78	0.434
	M	-0.014	-0.115	10.2	2.96	0.003
Gender	U	0.559	0.540	3.9	1.16	0.248
	M	0.560	0.486	14.9	4.25	0.000
BMI	U	0.030	-0.026	5.5	1.65	0.100
	M	0.029	0.082	-5.3	-1.45	0.147
Ethnicity	U	0.313	0.449	-28.3	-8.37	0.000
	M	0.314	0.329	-3.3	-0.97	0.331
Siblings	U	0.829	0.870	-11.3	-3.37	0.001
	M	0.830	0.807	6.5	1.72	0.085
Cadre rank	U	0.086	-0.074	16.0	4.78	0.000
	M	0.083	0.406	-32.3	-7.23	0.000
Home distance	U	0.077	-0.066	14.4	4.28	0.000
	M	0.075	0.112	-3.6	-1.02	0.307
Sociability	U	0.454	-0.390	92.8	27.64	0.000
	M	0.453	0.320	14.6	4.25	0.000
School type	U	0.766	0.792	-6.2	-1.85	0.065
	M	0.767	0.796	-6.9	-1.99	0.047
School quality	U	0.105	-0.090	19.5	5.80	0.000
	M	0.103	0.156	-5.3	-1.45	0.147
Intimacy with parents	U	0.174	-0.149	32.8	9.70	0.000
	M	0.173	0.062	11.3	3.20	0.001
Emotion regulation	U	0.267	-0.229	51.0	15.20	0.000
	M	0.267	0.187	8.2	2.14	0.032
Quality of life	U	0.605	-0.519	137.4	40.30	0.000
	M	0.604	0.608	-0.4	-0.16	0.877

Note. U=Unmatched, M=Matched
